# An immune cell spray (ICS) formulation allows for the delivery of functional monocyte/macrophages

**DOI:** 10.1038/s41598-018-34524-2

**Published:** 2018-11-02

**Authors:** Valerie Beneke, Fennja Küster, Anna-Lena Neehus, Christina Hesse, Elena Lopez-Rodriguez, Kathrin Haake, Anna Rafiei Hashtchin, Juliane Wilhelmine Schott, Dorothee Walter, Armin Braun, Willem F. Wolkers, Mania Ackermann, Nico Lachmann

**Affiliations:** 1 Institute of Experimental Hematology, Hannover Medical School, Hannover, Germany; 2 REBIRTH Cluster of Excellence, Hannover, Germany; 3 Fraunhofer Institute for Toxicology and Experimental Medicine (ITEM), Hannover, Germany; 4 Biomedical Research in Endstage and Obstructive Lung Disease (BREATH), German Center for Lung Research, Hannover, Germany; 5 Institute of Functional and Applied Anatomy, Hannover Medical School, Hannover, Germany; 6 Institute of Multiphase Processes, Leibniz Universität Hannover, Hannover, Germany

## Abstract

Macrophages are key cells of the innate immune system and act as tissue resident macrophages (TRMs) in the homeostasis of various tissues. Given their unique functions and therapeutic use as well as the feasibility to derive macrophages *in vitro* from hematopoietic stem cell (HSC) sources, we propose an “easy-to-use” immune cell spray (ICS) formulation to effectively deliver HSC-derived macrophages. To achieve this aim, we used classical pump spray devices to spray either the human myeloid cell line U937 or primary murine HSC-derived macrophages. For both cell types used, one puff could deliver cells with maintained morphology and functionality. Of note, cells tolerated the spraying process very well with a recovery of more than 90%. In addition, we used osmotic preconditioning to reduce the overall cell size of macrophages. While a 800 mosm hyperosmolar sucrose solution was able to reduce the cell size by 27%, we identified 600 mosm to be effective to reduce the cell size by 15% while maintaining macrophage morphology and functionality. Using an isolated perfused rat lung preparation, the combinatorial use of the ICS with preconditioned and genetically labeled U937 cells allowed the intra-pulmonary delivery of cells, thus paving the way for a new cell delivery platform.

## Introduction

Macrophages are hematopoietic cells of the myeloid lineage and represent important regulators of the innate immune system as well as key players in tissue homeostasis. Macrophages can be found in a multitude of organs (referred to as tissue resident macrophages; TRMs), for example as microglia in the brain, Langerhans cells in the skin, Kupffer cells in the liver, or as alveolar macrophages (AMs) in the lungs. Especially the latter are of great therapeutic interest, as AMs play an important role in lung tissue integrity by sensing pathogens, regulating immune responses and thereby contributing to tissue homeostasis, protection and repair^1^. It was believed for a long time that TRM populations are solely derived from circulating, bone marrow-derived monocytes. However, several recent publications employing genetic fate mapping tools elegantly demonstrate that a number of TRM populations arise early during hematopoietic development from progenitor cells in the yolk sac and fetal liver^2,3^. Thereafter, these early pre-macrophages seed the fetal tissues and adapt to the specific organ niche^4^. While most TRM populations possess stem cell-like features and are able to maintain their population under homeostatic conditions, also bone marrow-derived monocytes (BMDMs) can replenish resident macrophage pools in case of organ damage or disease. After infiltration of the respective organ, BMDMs are also able to adapt to the instructive tissue environment and gain the functional and transcriptional fingerprint of the resident macrophage population^5,6^. This exceptional, stem cell-like plasticity renders bone marrow-derived monocytes/macrophages an attractive target population for cell therapeutic approaches.

Given the important role of TRMs in organ homeostasis, macrophage dysfunction has been related to a variety of diseases. As an example, impairment of AMs has been shown to interfere with the surfactant metabolism, causing the rare pulmonary disease known as pulmonary alveolar proteinosis (PAP). The hereditary form of PAP (herPAP) is caused by mutations in the granulocyte-macrophage colony-stimulating factor (GM-CSF) receptor genes, resulting in disturbed alveolar macrophage development and function. As a consequence, herPAP patients suffer from massive protein accumulation in the lungs, and life-threatening respiratory insufficiency^7,8^. In addition to the development of herPAP, malfunctional AMs have also been associated with other respiratory diseases e.g. cystic fibrosis^9^. To establish a novel and cause directed therapy, we and others recently exploited the therapeutic potential of BMDMs as a novel cell-based treatment approach for herPAP. In these proof-of-concept studies, a single intra-pulmonary administration of stem cell-derived macrophages resulted in life-long therapeutic benefit in transplanted animals, thereby introducing a new concept of cell therapy using mature macrophages^10,11^.

To further translate the intra-pulmonary transplantation of macrophages into clinical practice, an “easy-to-use” cell transfer system is warranted. Here, a cell application system which would allow for a local cell administration, e.g. directly into the lung microenvironment, is of high therapeutic value as several studies have suggested superior effects of local compared to systemic administration of macrophages. With respect to clinical translation, the delivery of macrophages into the lung environment may be accomplished via the use of bronchoscopy instruments. This scenario however, represents a quite invasive process and requires general anesthesia.

Although bronchoscopy instruments are already regularly used in the clinics, we aim to establish an alternative and to provide a proof-of-concept study for an immune cell spray (ICS) formulation, which is able to locally deliver macrophages. Given the pre-clinical efficacy of intra-pulmonary macrophage transplantation in herPAP, an ICS may also be applied to deliver macrophages for the treatment of other pulmonary diseases. In addition to the pulmonary application, the development of an ICS would open a broad range of applications also for other tissues (e.g. ectopic use on skin). Indeed, a cell spray formulation has been applied in the past for the delivery of skin cells to burn wounds^12,13^. Given the important role of infiltrating bone marrow-derived monocyte/macrophages and resident Langerhans cells in wound healing and as a first line of cellular immunity, an ICS may also be used to deliver macrophages ectopically onto the skin in order to (i) support wound healing, (ii) combat/prevent wound infections, or (iii) reduce scar formation^14^.

With the objective to develop an ICS for the local administration of macrophages either onto the skin or directly into the lung environment, we demonstrate the efficient use of a classical pump spray device to spray myeloid cell lines as well as primary murine BMDMs. Importantly, the cells showed a high viability as well as maintained functionality after the spraying process. To further develop the ICS for the administration of macrophages into the bronchoalveolar space, we used hyperosmolar conditions to reduce the cell size of macrophages. In the present work, we were able to identify optimal conditions to reduce the cell size of macrophages while maintaining important functional characteristics. Moreover, we demonstrate the efficient local administration of shrunken cells into a perfused rat lung via a micro sprayer device, paving the way for macrophage cell spray formulations.

## Results

### Development of an immune cell spray (ICS) formulation

**Figure 1:**
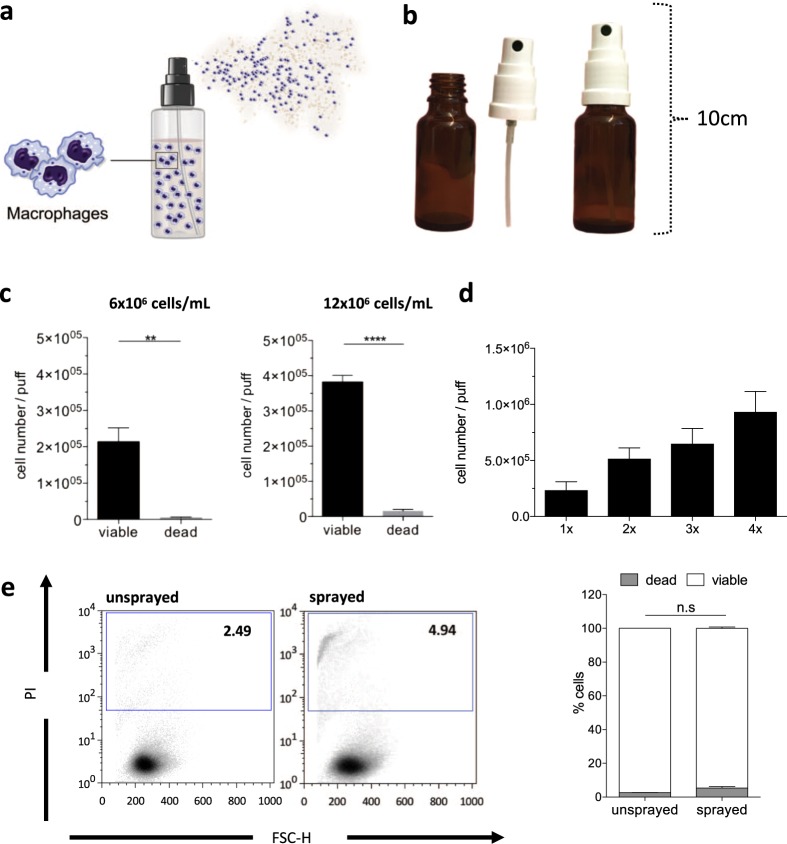
Spraying of U937 cells. (**a**) Schematic overview of the cell spraying procedure and **(b)** image of the pump spray devices used for our studies. (**c**) Numbers of viable and dead U937 cells after the spraying process, analyzed via trypan blue staining. Initial concentrations of 6 × 10^6^ cells/mL (left) and 12 × 10^6^ cells/mL (right) were used (significance of **P < 0.01 or ****P < 0.0001 by two-tailed paired student t-test, n = 3–6 mean ± SEM). (**d**) Cell numbers/puff at a cell concentration of 6 × 10^6^ cells/mL (n = 6, mean ± SEM). **(e)** Evaluation of cell viability after the spraying process by propidium iodide (PI) staining (Left: Representative flow cytometry analysis. Right: Quantification of cell viability n = 3 mean ± SEM). (n.s: denotes not significant. Statistical analysis: analyzed by two-way repeated measurements (RM) ANOVA with Bonferroni post-hoc-test).

In order to develop an immune cell spray (ICS) formulation, we used classical pump spray devices (Fig. 1a,b) and investigated the feasibility to spray either human hematopoietic cell lines or primary murine bone marrow-derived macrophages. In a first set of experiments, we used the human myeloid cell lines K562 or U937 and tested a variety of spraying devices for their applicability to deliver myeloid cells. With respect to practicability, sterility and cleaning procedures of the individual devices, we identified a classical pump spray/nozzle configuration (Fig. 1b) (brown glass, volume 20 mL, spray volume approx. 50 μL, company Rixius AG, Mannheim Germany) to be most promising. This spray device has been used for further studies.

In order to evaluate the viability of U937 cells after the spraying process, at least 1 mL of a cell solution containing 6 or 12 × 10^6^ cells/mL was filled into the spray device and then sprayed into a falcon tube. Using a cell density of 6 × 10^6^ cells/mL, 2.1 × 10^5^ ± 3.8 × 10^4^ or 3.2 × 10^5^ ± 1.9 × 10^4^ (mean ± SEM) viable cells per puff could be detected for U937 or K562 cells respectively (Fig. 1c and Supp. Fig. 1a). After increasing the concentration of the cell solution to 12 × 10^6^ cells/mL approximately twice as many cells per puff could be detected for both cell types (Fig. 1c and Supp. Fig. 1a). To further assess the dynamics of the spraying process, we quantified cell numbers after repetitive spraying of U937 and K562 cell solutions, showing that the number of cells rise proportionally with each puff (Fig. 1d and Supp. Fig. 1b). Of note, given a spraying volume of 42.23 ± 7.2 µL (mean ± SEM), the expected cell dose would range approx. 3 × 10^5^ cells/puff for a cell suspension containing 6 × 10^6^ cells/mL. As a next step, we evaluated the cell viability of sprayed cells. Here, irrespective of the cell density used, we observed an overall viability of >90% of sprayed cells (97.97 ± 0.34% and 96.44 ± 1.06% for U937 cells, 6 and 12 × 10^6^ cells/mL, respectively and 96.00 ± 1.53% and 99.09 ± 0.28% for K562 cells, 6 and 12 × 10^6^ cells/mL, respectively, mean ± SEM) (Fig. 1c and Supp. Fig. 1a). The viability of U937 cells following the spraying process was further confirmed by the detection of apoptotic cells using propidium iodide (PI) staining. Using flow-cytometric analysis, we could not detect any significant differences in the percentage of PI^+^ cells before and 1 hour after the spraying process (Fig. 1e).

### Delivery of macrophages as a cell spray formulation does not influence viability or functionality

**Figure 2:**
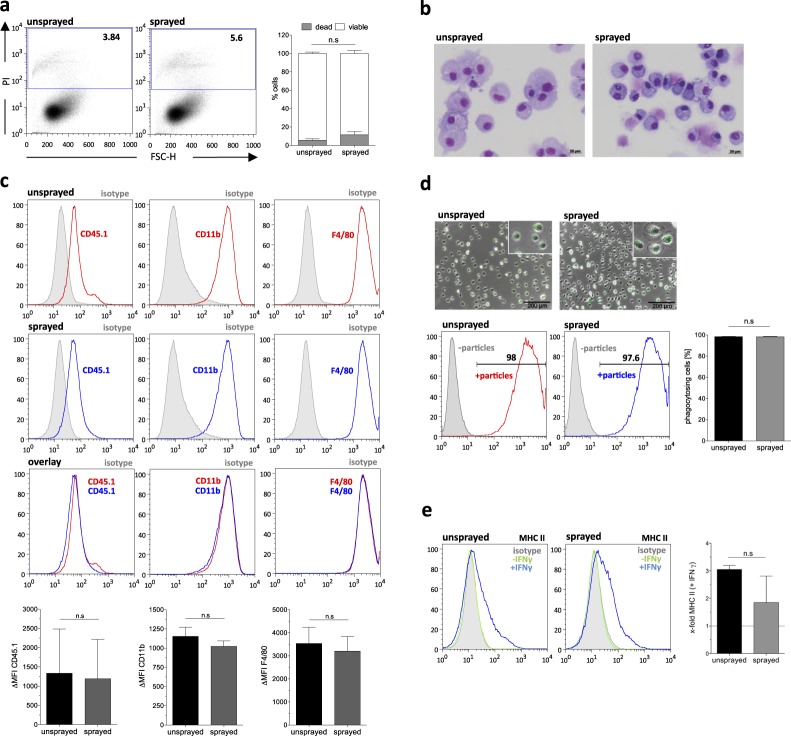
Functionality of bone marrow (BM)-derived macrophages after spraying. (**a**) Flow cytometric analysis of cell viability by propidium iodide (PI) staining of unsprayed and sprayed BM-derived macrophages (Left: representative images, right: quantification of n = 3, mean ± SEM). (**b**) Papenheim staining of cytospin preparations of sprayed and unsprayed BM-derived macrophages (scale bar: 20 µm) (**c**) Flow cytometric analysis of CD45.1, CD11b and F4/80 expression of unsprayed and sprayed BM-derived macrophages. Overlays of representative histograms; grey filled line: isotype, red/blue line: surface marker of unsprayed or sprayed macrophages. Lower panels: Delta mean fluorescent intensity (MFI) of surface marker expression of unsprayed and sprayed BM-derived macrophages (delta MFI was calculated by subtracting isotype (negative) MFI from surface marker (positive) MFI; n = 3, mean ± SEM). (**d**) Phagocytic activity of unsprayed and sprayed BM-derived macrophages. Upper panels: representative fluorescent microscopy images of BM-derived macrophages 2 hours after incubation with pHRodo *E. coli* BioParticles (scale bar: 200 µm). Lower panel: Representative flow cytometric analysis (grey filled line: cells without *E. coli* BioParticles, red line: unsprayed cells with *E. coli* BioParticles, blue line: sprayed cells with *E. coli* BioParticles) and quantification of n = 3 (mean ± SEM). (**e**) Analysis of MHC-II surface marker expression by flow cytometry of unsprayed and sprayed BM-derived macrophages before and after stimulation with IFNγ. Left: Representative histograms (grey filled line: isotype, green line: non-stimulated control, blue line: IFNγ stimulated) and right: Quantification of the fold change in MFI, n = 3, mean ± SEM). (n.s: denotes not significant. Statistical analysis: **a**: two-way repeated measurements (RM) ANOVA with Bonferroni post-hoc-test; (**c**–**e**) two-tailed paired student t-test).

After we successfully established the ICS, we next evaluated the possibility to spray macrophages which have been previously differentiated from murine bone marrow. Similar to the afore mentioned studies, we first concentrated on the feasibility to spray bone marrow-derived macrophages (BMDMs) (3 × 10^6^ cells/mL) and analyzed their phenotype and functionality before and after the spraying process. Using the same spray device, we observed a high viability of primary macrophages 1 hour after the spraying process, which was very similar to non-sprayed control cells (Fig. 2a). Moreover, we confirmed a macrophage like phenotype of the sprayed macrophages by cytospin staining as well as flow cytometric analysis (Fig. 2b,c). Of note, evaluating different cytospin preparations (n = 3), we could observe a reduction in the size of macrophages by approx. 21% following the spraying process compared to the non-sprayed counterparts. Following the spraying process, macrophages maintained typical surface markers and stained positive for CD45.1, CD11b, and F4/80 (Fig. 2c). Even more importantly, we could also see maintained functionality of macrophages after the spraying process. Using a pHrodo labeled *E. coli* bioparticles phagocytosis assay, macrophages after the spraying process were able to phagocytose bioparticles as efficient as non-sprayed control cells (Fig. 2d). Moreover, the spraying process had no influence on the ability of macrophages to upregulate MHCII in response to IFNγ stimulation (Fig. 2e).

### Macrophage cell size reduction by applying hyperosmolar sugar solutions

Having proven the feasibility of the ICS, we next aimed to further optimize the macrophage spray formulation to make it suitable for the delivery of cells directly into the airways. Given the considerable large size of macrophages (approx. 15 µm diameter)^15^, we investigated the effect of hyperosmolar sucrose solutions to temporary reduce the overall cell size of macrophages. The reduction of the cell size is of particular importance as the size of particles has been shown to be a crucial parameter for intra-pulmonary drug delivery^16^.

**Figure 3:**
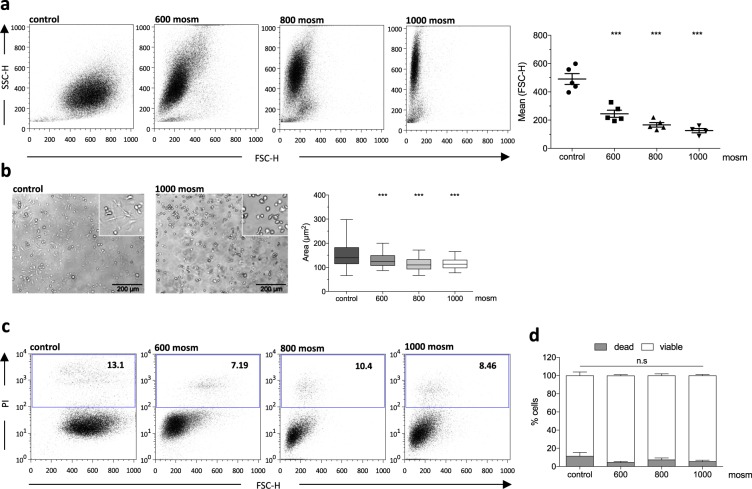
Shrinking of BM-derived macrophages using hyperosmolar solutions. (**a**) Flow cytometric analysis using FSC-H/SSC-H plot discrimination (left panel) and mean of FSC-H (right panel) for control (PBS) and hypertonic solutions (n = 4–5, biological repeats, mean ± SEM, significance of ***P < 0.001 by one-way ANOVA with Tukey’s post-hoc-test). (**b**) Representative brightfield images of BM-derived macrophages after incubation in control medium and 1000 mosm solution (scale bar: 200 µm) and ImageJ analysis of cell area (right panel; n = 105–482, technical repeats, mean with 95% CI, significance of ***P < 0.001 by one-way ANOVA with Tukey’s post-hoc-test). (**c**) Flow cytometric analysis of cell viability by propidium iodide (PI) staining of macrophages after incubation (60 min) in control (PBS) and hypertonic solutions. (**d**) Quantification of cell viability after incubation in control (PBS) and hypertonic solutions (n = 4–5 mean ± SEM). (n.s: denotes not significant analyzed by two-way ANOVA with Bonferroni post-hoc-test).

When applying hyperosmolar D(+)-sucrose solutions (600, 800 and 1000 mosm) to murine BMDMs or U937 cells, we observed morphological changes already within 15 min after incubation as indicated by a change in forward/sideward scatter (FSC/SSC) properties (Fig. 3a and Supp. Fig. 2a). Of note, the decrease in FSC values was concentration dependent, indicating a reduction of cell size most likely explained by the osmotic efflux of water (Fig. 3a). The same observation could be made in treated U937 cells, showing the maximal osmotic effect using 1000 mosm sucrose solutions (Supp. Fig. 2a). To further verify this observation, we measured the size of treated cells after 1 hour in hyperosmolar solutions by light microscopy and subsequent ImageJ analysis. Similar to the flow cytometry data, we could observe a significant decrease of the cell area in all three hyperosmolar conditions of primary murine BMDMs (Fig. 3b) as well as U937 cells (Supp. Fig. 2b). Applying sucrose solutions of 600, 800, or 1000 mosm to BMDMs, we observed a reduction in the cell area by 15.5%, 26.9%, and 26.0% (control: 155.7 µm^2^, 600 mosm: 131,6 µm^2^, 800 mosm: 113,9 µm^2^, 1000 mosm: 115.2 µm^2^; all mean values), respectively. Similarly, preconditioning of U937 cells led to cell size reduction of 17.1%, 27.4% and 34.8% (control: 113.8 µm^2^, 600 mosm: 94,34 µm^2^, 800 mosm: 82,63 µm^2^, 1000 mosm: 74.26 µm^2^; all mean values) respectively (Fig. 3b and Supp. Fig. 2b). Importantly, treated murine BMDMs as well as U937 cells tolerated the incubation in hyperosmolar solutions very well indicated by less than 12% dead cells after 60, or 15 minutes of incubation, respectively (Fig. 3c,d and Supp. Fig. 2c,d).

### Murine BMDMs remain functionally and phenotypically normal following temporary shrinking

**Figure 4:**
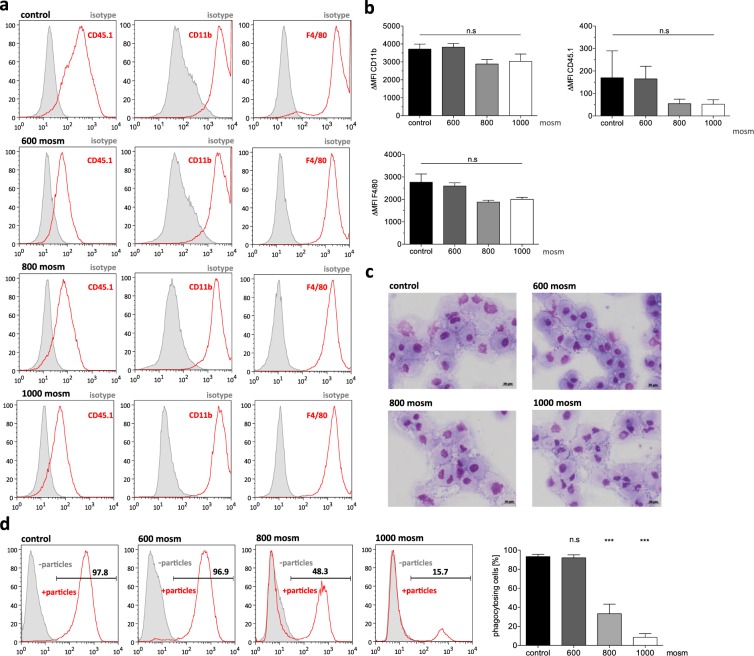
Functional analysis of BM-derived macrophages after incubation in hyperosmolar solutions. (**a**) Flow cytometric analysis CD45.1, CD11b and F4/80 surface marker expression on BM-derived macrophages after incubation in control (PBS) and hypertonic solutions (grey filled line: isotype, red line: surface marker). (**b**) Delta mean fluorescent intensity (MFI) of surface marker expression (delta MFI was calculated by subtracting isotype (negative) MFI from surface marker (positive) MFI; n = 3 biological repeats, mean ± SEM, n.s: not significant by one-way ANOVA with Tukey’s post-hoc-test). (**c**) Cytospins of BM-derived macrophages after incubation in control (PBS) and hypertonic solutions (scale bar: 20 µm). (**d**) Phagocytic activity of unsprayed and sprayed BM-derived macrophages. Left: Representative flow cytometric analysis (grey filled line: cells without *S. aureus* BioParticles, red line: cells with *S. aureus* BioParticles) and quantification of n = 3 (mean ± SEM) (n.s denotes not significant, significance of ***P < 0.001 by one-way ANOVA with Tukey’s post-hoc-test).

After demonstrating efficient size reduction of primary murine BMDMs and U937 cells by incubation in hyperosmolar sugar solution, we evaluated the phenotype and functionality of the treated BMDMs. This is of great importance, as maintained functionality of shrunken macrophages would be a prerequisite for future clinical translation. Flow cytometric analysis revealed a maintained surface marker profile of CD45.1^+^/CD11b^+^/F4/80^+^ 1 hours post incubation in 600, 800 or 1000 mosm hypertonic solutions. Moreover, no significant change in mean fluorescent intensity (MFI) values could be observed, indicating no effect on cell surface marker expression (Fig. 4a,b). Of note, when cells have been pretreated in hypertonic solutions and subsequently be maintained in 300 mosm physiological conditions, a normal characteristic cell morphology as well as normalized overall cell size was observed (Fig. 4c). We next evaluated the phagocytic capacity of cells that were first cultured in hypertonic solutions and then treated with physiological conditions to become normal. In these assays, we observed efficient und steady phagocytosis for cells previously shrunk in a 600 mosm sugar solution. However, BMDMs incubated in higher concentrated sugar solutions (800 mosm and 1000 mosm) showed an impaired phagocytic potential, indicating a detrimental effect on the functionality by the hyperosmolaric treatment (Fig. 4d). Primary BMDMs also tolerated exposure to hyperosmolaric solutions (600 mosm) followed by the spraying process. The so treated BMDMs were able to efficiently phagocytose *E. coli* bioparticles equally as efficient as BMDMs cultured in control physiological conditions (Supp. Fig. 3).

### Local administration of GFP-labeled U937 cells into isolated perfused rat lungs

To further prove our ICS concept to locally deliver monocyte/macrophages directly into the airways, we have performed proof-of-concept studies utilizing a Microsprayer® Aerosolizer device for rats (model IA-1B-R, PennCentury) in combination with the isolated perfused rat lung (IPL) model^17^ (IPL-2, Harvard Apparatus, Holliston, MA, USA). In order to visualize the cells after transplantation, we employed genetically labeled U937 cells previously transduced with lentiviral vectors expressing GFP from a CMV early enhancer/chicken β-actin (CAG) promoter (kindly provided by Daniela Paasch, Hannover Medical School).

**Figure 5:**
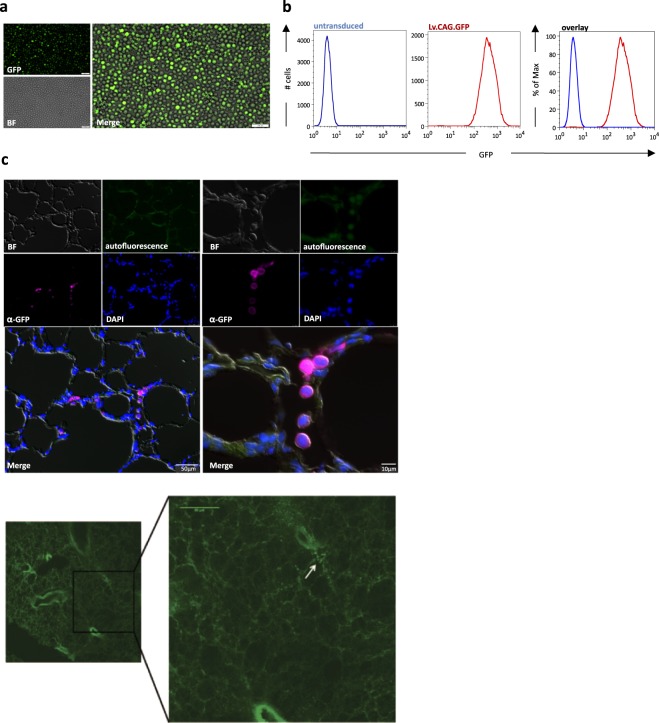
Local administration of GFP-labeled U937 cells into isolated perfused rat lung (IPL) (**a**) Representative fluorescent microscopy images of U937 cells transduced with lentiviral vectors expressing GFP from a CMV early enhancer/chicken beta-actin promoter (Lv.CAG.GFP) (scale bar: 100 µm; BF: brightfield) (**b**) Flow cytometric analysis of GFP expression in Lv.CAG.GFP transduced U937 cells (blue line: untransduced cells, red line: cells transduced with Lv.CAG.GFP) (**c**) Analysis of U937 cell localization after application to the IPL. Upper panels: Immunofluorescence staining of cryostat sections generated from the lobus accessorius of the IPL (left: scale bar 50 µm, right: scale bar 10 µm), U937 cells were detected by anti-GFP antibody staining. Lower panel: Representative images of horizontal 2-Photon microscopy of lobus accessorius (right: scale bar: 60 µm).

To ensure efficient tracking, we first confirmed strong GFP expression in the transgenic U937 cells (Fig. 5a,b). In order to reach the lower airways in our IPL model, we subjected the transduced U937 cells to hyperosmolaric preconditioning using the afore evaluated 600 mosm sugar solution. As a next step, we applied a cell solution of 4 × 10^6^ cells/300 µL directly into the trachea of the rat IPL using the Microsprayer device. During and directly after cell administration, a total of 10 deep inspiratory breaths were performed in order to ensure macrophage deposition into distal airway spaces. Respiratory parameters were evaluated simultaneously and revealed a short-term reduction of airflow, which was reflected in a slight increase in lung resistance (R_L_). This increase was restored to baseline levels after performance of the deep breaths. In addition, a reduction of dynamic lung compliance (C_dyn_) by 75% was found. These effects could not be reversed by performance of the deep breaths and lasted throughout the subsequent experimental time of 1 hour. Using the afore mentioned technique, a cleared air-inflated and fixed lobus accessorius was observed in a scanning horizontal 2-Photon microscope where cell-like bright green structures can readily be localized in alveolar spaces (Fig. 5c). A closer look using specific fluorescence immunostaining against GFP revealed GFP-positive cells (of approx. 8–10 µm diameter) not only at the air-spaces but also in close contact with alveolar epithelium (Fig. 5c). Therefore, cells appear to reach the alveolar distal spaces of the lungs.

## Discussion

In summary, we introduce an immune cell spray (ICS) formulation which would allow for the local delivery of macrophages to skin or lung respectively. We demonstrate that different myeloid cell lines as well as primary murine BMDMs can be delivered as a cell spray employing classical pump spray devices, survive the spraying process, and remain functional afterwards.

The so developed ICS formulation could in principle be applied to supply macrophages into different tissues e.g. into the lung environment or onto skin wounds. Indeed, cell spray formulations delivering skin cells to burn wounds were already able to prove feasibility, demonstrating profound clinical effects while reducing the amount of donor skin needed per wound area^18,19^. Given the regulatory role of monocyte/macrophages and Langerhans cells in wound healing as well as tissue homeostasis, the supplementary use of macrophages in such cell spray compositions might be of advantage. Alternatively, macrophages also represent an important part of the innate immune system with strong antimicrobial activities^20^. Thus, a macrophage-based cell spray formulation may be used in wound healing and to counteract bacterial infections. Bacterial infections associated with burn wounds, represent a clinical challenge and can cause severe clinical complications^21^. This becomes even more problematic if the pathogens are refractory to current standard antibiotic therapy. In such a clinical scenario, a combined therapeutic approach using a cell spray formulation of macrophages and fibroblasts/keratinocytes together with current care medicine may be beneficial and might offer new perspectives to fight infections and support wound healing. Following this hypothesis, a recent study already investigated a monocyte/macrophage-based cell therapy applying macrophages via a biomimetic hydrogen scaffold to cutaneous wounds. Using M-CSF-derived bone marrow macrophages, the authors could demonstrate accelerated wound healing in wild type as well as diabetic mice^22^.

In addition to the use of a macrophage-based ICS formulation to improve wound healing, the envisioned ICS may also be used to locally apply macrophages directly into the lung environment. In contrast to the administration of macrophages onto the skin, a direct pulmonary transfer of cells may require a smaller size of the cells in order to reach the small bronchioles and alveoli. Considering the lung architecture, the upper respiratory tract with the nasopharynx, trachea, and large bronchi is responsible for the filtration and removal of 70–90% of particles^16^. To circumvent the clearance of cells and to further reach the lower respiratory tract, the envisioned size of the particles/cells should ideally be less than 5 µm, if applied by oral inhalation^16,23,24^. In our studies, we reached a cell size of <10 µm for U937 and approx. 12 µm for BMDMs, however we did not reach the critical 5–8 µm size. Here, more intensified studies with improved protocols for cell shrinking using hyperosmolar sugar solutions or even salt solutions have to be performed.

Given our ICS proof-of-concept study, short term bronchoscopy intervention or even endotracheal intubation may be combined with the use of macrophages in order to deliver the cells into the lower airways and to establish a clinically applicable cell administration procedure. While macrophages are generally considered to be of 13–20 µm (in suspension)^15,25^, we here evaluated the incubation of macrophages in a hyperosmolar sugar solution as a method to reduce cell size. To achieve this aim, we used a sucrose gradient to gently reduce cell size. Cell volume response to anisotonic solutions can be described by the Boyle-van’t Hoff law. However, cells only respond as linear osmometers in a defined osmotic range within the osmotic tolerance limits of the cell^26^. Applying different sucrose solutions with 600, 800, or 1000 mosm, we were able to efficiently reduce the cell size by 15–26% for BMDMs or 17–35% for U937 cells (calculated to the initial size). Although we used a hypertonic sucrose solution instead of a salt solution to keep the stress to the cells as low as possible, we observed an impairment in functionality after incubation of primary macrophages with 800 and 1000 mosm. However, when applying the 600 mosm solution we observed a significant size reduction while maintaining the functionality of the cells. It could well be that with shorter exposure times, cells may be able to survive exposure to higher hyperosmolar solutions while maintaining their functionality. Although we performed proof-of-concept experiments demonstrating an efficient spraying of previously shrunk cells (600 mosm) into a rat lung explant culture, it remains to be elucidated whether the reduction in cell size would be sufficient to allow the cells to reach the lower respiratory tract. In our explanted rat perfusion lung model, we directly applied the cells into the upper airways via a microsprayer device, bypassing the nasopharynx section. While our studies represent a proof-of-concept study, further experiments which would address the feasibility of an e.g. cell nebulizer would be warranted to investigate nasal vs. oral aerosol inhalation. While the process of spraying also has an effect on macrophages alone (determined by cytospin preparations), the combinatorial effect of spraying and shrinking of macrophages needs to be investigated in more detail. Of note, these values are derived from cytospin preparations and therefor might not represent the actual cell size of living cells in suspension. As an alternative method to the temporal hyperosmotic preconditioning, also the use of freeze-drying preservation methods might be applied. Although freeze-drying of mononuclear cells has been proven feasible^27,28^, further improvements have to be made with respect to cell viability before using macrophages. Similarly, also further improvements in the cell delivery techniques have to be made. In our proof-of-concept study we have used a pump and nozzle configuration, which might not allow for the production of droplets in appropriate sizes for uniform lung deposition. As an alternative, pressurized metered-dose inhalers (pMDIs), “dry powder” inhalers (DPIs), or even nebulizers^29^ could be combined with modified macrophages in order to develop a clinically applicable ICS.

In order to establish the proof-of-concept for the ICS, we used primary macrophages from murine bone marrow. While use of the ICS in combination with murine cells has been proven feasible, human macrophages have to be investigated next. Whereas an allogeneic or even an autologous approach (e.g. a hematopoietic stem cell (HSC) based gene therapy combined with a macrophage-differentiation approach) might be an option, a promising alternative is represented by the use of human induced pluripotent stem cell (iPSC) technology. Previously, different studies have already proven the generation of macrophages from human and murine iPSC^30–32^. Human iPSC macrophages demonstrate high phenotypical and functional similarities to BMDMs and, even more important, can engraft into the lung in humanized mouse models and gain the transcriptional and functional fingerprint of the resident population^30,33^. Further studies employing human macrophages, which are either generated from adult type HSC or pluripotent stem cells in combination with the ICS may allow for new cell-based therapies and to apply macrophages as an off the shelf cell-product.

## Methods

### Cell culture

The human cell lines U937 and K562 were cultured in RPMI 1640 (Thermo Fisher Scientific, Waltham, MA, USA) supplemented with 10% fetal calf serum (FCS, Biochrom, Berlin, Germany) and 1% penicillin/streptomycin (Thermo Fisher Scientific).

### Isolation of murine bone marrow and macrophage differentiation

The procedure of isolation of murine bone marrow/rat lung were in accordance with the German Animal Welfare Legislation (§4, TierSchG) and approved by the local Institutional Animal Care and Research Advisory Committee and permitted by the Lower Saxony State Office for Consumer Protection and Food Safety. While no experiments on living animals was performed, no approval from the local ethical committee was needed, which is in accordance with institutional guidelines. For isolation and cultivation of murine bone marrow-derived macrophages, femur, tibiae of B6.SJL-*Ptprc*^*a*^ *Pepc*^*b*^/BoyJ (CD45.1) (purchased from animal facility ZTL Hannover Medical School) mice were flushed with PBS supplemented with 2% FCS and 0.5% EDTA (Omnilab, Gehrden, Germany) and centrifuged at 500 x g for 5 minutes. Lysis of erythrocytes was performed using red cell lysis buffer (156 mM NH_4_Cl, 46 mM KHCO_3_, 0.5 mM EDTA) for 2 minutes. Purified precursor cells were cultivated and differentiated in RPMI 1640 + 30% L929-cell conditioned medium (LCCM) as a source for macrophage colony-stimulating factor for 7 days.

### Isolated Perfused Rat Lung model (IPL)

To assess the deposition of macrophages in the lower respiratory tract, an isolated perfused rat lung (IPL) model was used. As a donor animal, a female wistar rat (Crl:Wi (Han)) (Charles River Laboratories in Sulzfeld, Germany) was used. The IPL was performed as previously described by Uhlig (Uhlig, S. (1998) “*The isolated perfused rat lung. Methods in Pulmonary Research. 1st, pp. 29–55, Basel, Birkhäuser*)” and Fischer *et al*.^17^. In brief, isolated lungs (materials during isolation were perfused in a recirculating system with constant pressure (12 cmH_2_O). This resulted in a flowrate of 27.8 mL/min. Ventilation was switched from positive to negative pressure (end-inspiratory: −7.5 cm H_2_O; end-expiratory: −3.0 cm H_2_O; inspiratory oxygen fraction FiO_2_ = 0.21) after the isolated organ was placed into the artificial thoracic chamber. Ventilation frequency was set to 80 breaths/min and the ratio of inspiratory to expiratory duration to 1:1. An equilibration period of 30 min was provided before administration of the cells. Every 5 minutes, a hyperinflation (21 cm H_2_O) was performed to re-open atelectatic lung areas and enhance release of surfactant.

### Spraying of cells

Spraying of cells was performed utilizing a pump spray bottle made out of glass (brownglass, volume 20 mL, white plastic finger sprayer cap, spray volume approx. 50 μL company Rixius AG, Mannheim Germany). One mL of cell suspension was transferred into the flask to ensure effective spraying. The cell suspension was sprayed into a 15-mL falcon tube in multiple spraying rounds to yield sufficient cell numbers for following analysis. Evaluation of cell number and cell viability after spraying was performed using trypan blue (Thermo Fisher Scientific) exclusion.

### Exposure to hyperosmolar solutions

To shrink the cells, hypertonic solutions ranging from 600 mosm to 1000 mosm were prepared by adding the corresponding amount of D(+)-saccharose to isotonic PBS (300 mosm). For osmolarity experiments, 5 × 10^5^ cells were incubated in 2 mL of hypertonic solutions for 15 minutes or 1 hour to examine cell viability, FACS analysis and phagocytosis, respectively.

### Flow Cytometric Analysis

Analysis of surface marker expression was performed as described before^30^. For antibody staining of shrinked cells, cells were kept in hypertonic solutions supplemented with 2% FCS. Antibodies were used as follows: CD11b-PE (12-0112-82), CD45.1-APC (17-0453), F4/80-APC (17-4801-80), MHC-II (I-A/I-E)-APC (17-532-81), isotype controls: rat IgG2ak-APC (17-4321-41), mouse IgG2bk-PE (12-4031-81) (all from eBioscience). Propidium iodide (PI; Sigma-Aldrich, St. Louis, MO, USA) was diluted 1:100 and added directly before FACS analysis.

For measurement of MHC Class II upregulation after IFNγ stimulation macrophages were either left unstimulated or stimulated with 25 ng/mL IFNγ for 24 hours. Flow cytometry was performed using FACSCalibur (BD) and further analysed using FlowJo V8 (Tree Star).

### Cytospins

For cytospins, 3 × 10^4^ cells were centrifuged on object slides for 7 minutes at 600 x g and dried overnight. Slides were stained in May-Grünwald staining solution (0.25% (w/v) in methanol) for 5 minutes, followed by 20 minutes in 5% of Giemsa azur-eosin-methylene blue solution (0.4% (w/v)) in methanol, working solution was 0.02%) and washed extensively in aqua dest.

### Phagocytosis assay

For assessment of phagocytosis, 1 × 10^5^ cells were seeded in 12-well plates in standard medium. After >12 hours of settling, cells were incubated for 2 hours with pHRodo Green *E. coli* or pHRodo Red *S. aureus* BioParticles (Thermo Fisher Scientific) at a concentration of 1:20. Phagocytosis was performed in phenol-free RPMI 1640 (Thermo Fisher Scientific) supplemented with 10% FCS, 2% HEPES (AppliChem, Darmstadt, Germany), 1% L-glutamine (Invitrogen, Darmstadt, Germany) and 10 pg/mL murine M-CSF (R&D Systems, Minneapolis, MN, USA). Subsequently, cells were washed extensively, and the amount of incorporated BioParticles was evaluated by flow cytometry.

### ImageJ analysis

Determination of cell size was carried out using ImageJ2 “Particle Analysis”^34^. Monochrome pictures were taken and a manual threshold was set to separate the cells apart from the background. For particle analysis, minimum and maximum cell area as well as roundness values were selected manually to include as many single cells as possible.

### Statistical analysis

Statistical analysis was performed using Prism V6 (GraphPad, La Jolla, CA, USA). Paired statistics were used to analyze sprayed versus unsprayed conditions (paired student´s t-test and repeated measurements (RM) two-way analysis of variance (two-way ANOVA) with Bonferroni post-hoc-test). In all other cases one-way analysis of variance (one-way ANOVA) with Tukey’s post-hoc-test was performed, unless noted otherwise.

## Electronic supplementary material

Supplementary information

## Data Availability

The datasets generated during and/or analyzed during the current study are available from the corresponding author on reasonable request.
