# Supplementary material for: An immune cell spray (ICS) formulation allows for the delivery of functional monocyte/macrophages
Source: Sci Rep. 2018 Nov 2;8:16281. doi: 10.1038/s41598-018-34524-2 (PMC6214992; doi:10.1038/s41598-018-34524-2)
Supplement: Supplementary file 1 — Supplementary information [file 41598_2018_34524_MOESM1_ESM.pdf]

Title: **An immune cell spray (ICS) formulation allows for the delivery of functional monocyte/macrophages**

Running title: A myeloid cell spray formulation

Authors: *Valerie Beneke[1,2], *Fennja Küster[1,2], Anna-Lena Neehus[1,2], Christina Hesse[2,3,4], Elena Lopez-Rodriguez[2,4,5], Kathrin Haake[1,2], Anna Rafiei Hashtchin[1,2], Juliane Wilhelmine Schott[1], Dorothee Walter[2,3,4], Armin Braun[2,3,4], Willem F. Wolkers[2,6], Mania Ackermann[1,2], ⁾Nico Lachmann[1,2]

Affiliations: [1]Institute of Experimental Hematology, Hannover Medical School, Hannover, Germany
[2]REBIRTH Cluster of Excellence
[3]Fraunhofer Institute for Toxicology and Experimental Medicine (ITEM)
[4]Biomedical Research in Endstage and Obstructive Lung Disease (BREATH), German Center for Lung Research, Hannover, Germany
[5]Institute of Functional and Applied Anatomy, Hannover Medical School, Germany
[6]Institute of Multiphase Processes, Leibniz Universität Hannover, Germany

*These authors contributed equally to the work

Supplementary Information

Supplementary Figure S1

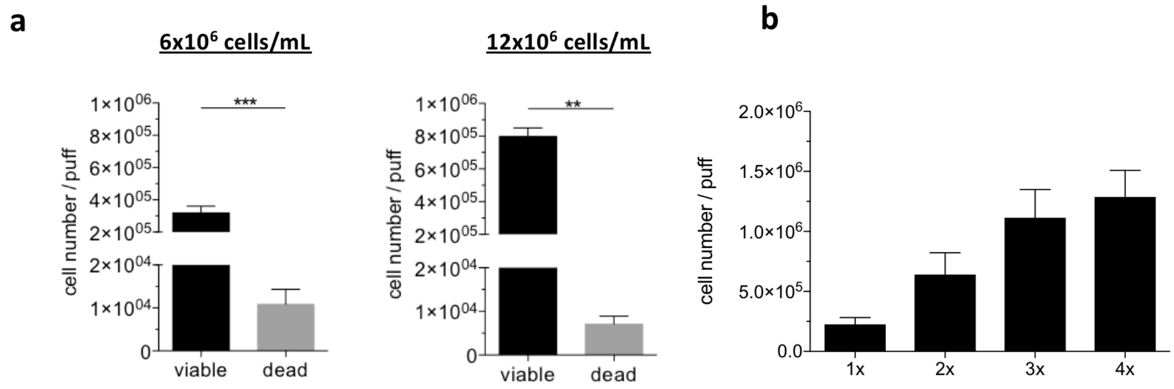

Figure S1. Spraying of K562 cells. (**a**) Numbers of viable and dead K562 cells after the spraying process, analyzed by trypan blue staining. Initial concentrations of 6x10^6 cells/ mL (left) and 12x10^6 cells/mL (right) were used (*significance of ** $P < 0.01$ or *** $P < 0.001$ by two-tailed paired student t-test). (**b**) Cell numbers/puff at a cell concentration of 6x10^6 cells/mL (n=3-6, mean ± SEM).

Supplementary Figure S2

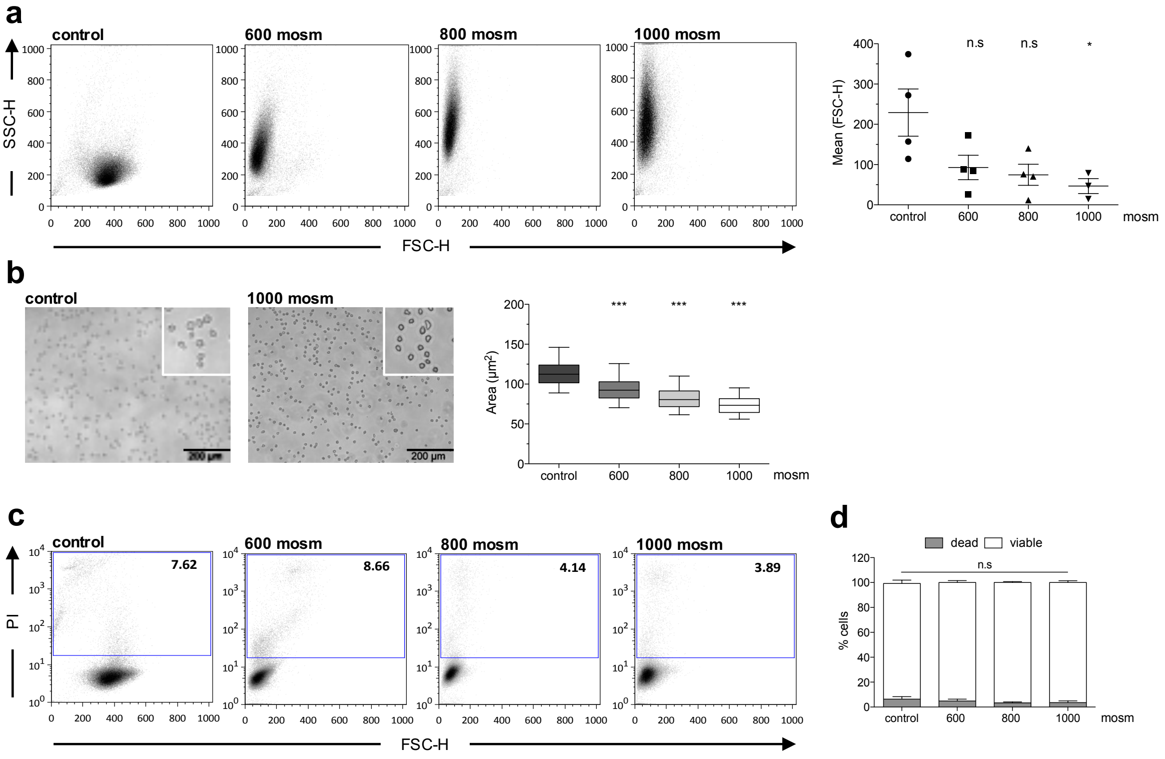

Figure S2 Shrinking of U937 cells using hypertonic solutions (**a**) Flow cytometric analysis of FSC-H/SSC-H plot (left) and mean of FSC-H (right) for control (PBS) and hypertonic solutions (n=3-4, biological repeats, mean ±SEM, n.s: not significant, *significance of $P < 0.05$ by one-way ANOVA with Tukey's post-hoc-test). (**b**) Representative brightfield images of U937 cells after incubation in control (PBS) and 1000 mosm solution (scale bar: 200 µm) and ImageJ analysis of cell area (right; n=426-926, technical repeats, mean with 95% CI, ***significance of $P < 0.001$ by one-way ANOVA with Tukey's post-hoc-test). (**c**) Flow cytometric analysis of PI incorporation in cells after incubation (15min) in control (PBS) and hypertonic solutions. Percentages state amount of PI positive cells compared to unstained cells. (**d**) Quantification of U937 cell viability after incubation in control (PBS) and hypertonic solutions (n=3-4, biological repeats, mean ±SEM). (n.s: denotes not significant analyzed by two-way ANOVA with Bonferroni post-hoc-test).

Supplementary Figure S3

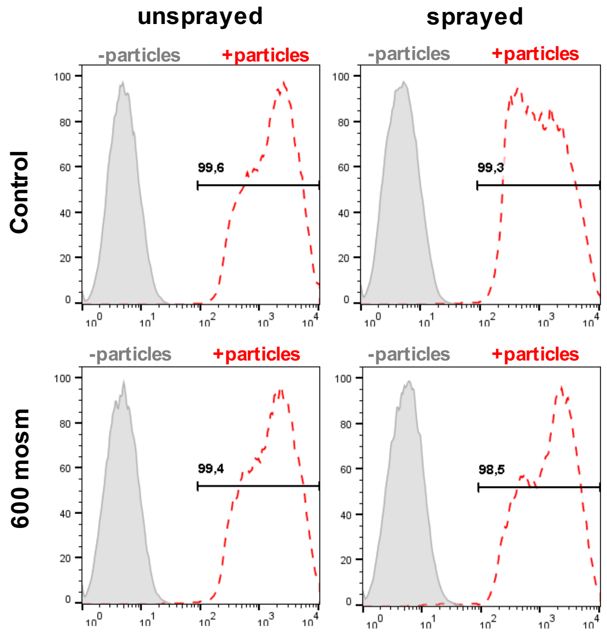

Figure S3. Phagocytic potential of BMDMs after combined shrinking and spraying. Non-treated BMDMs were analyzed for their phagocytic potential of *S. aureus* bioparticles under non-sprayed or sprayed condition only (upper panels). BMDMs treated in 600mosm hypertonic solutions were analyzed for their phagocytic potential of *S. aureus* bioparticles under non-sprayed or sprayed condition (lower panels). (grey filled curve: without bioparticles, red curve: treated with bioparticles).
